# Remarks on the Use of the Nux Vomica in Dysentery

**Published:** 1782

**Authors:** 


### SECTION II.

### ESSAYS AND OBSERVATIONS.

I. *Remarks on the uſe of the Nux vomica in Dyſentery. By* J. O. Hagſtröm, M. D. *Provincial Phyſician in Weſt-Gothland. Tranſlated from the Swediſh by a Member of the Society. Read* March 4, 1782.

PUTRID fevers and dyſenteries have of late prevailed much in this province, eſpecially among the lower ſort of people, many of whom have died before any proper remedies could be adminiſtered. Aged perſons and children ſuffered much under theſe complaints, but none more than ſcorbutic and conſumptive patients, very very few of the latter having been preferved, and thofe not without great trouble.

The dyfentery began to make its appearance in the autumn of 1772, and foon became very general, not only in the town of Linkoping, but likewife in the country. Having received orders from Baron Strömfield, our Governor, to vifit the different parifhes in which the epidemic prevailed, I prefcribed fuch remedies as are ufually recommended in cafes of this fort; but finding that they did not always produce the defired effect, and that the price of them oftentimes exceeded the abilities of the poor, I was defirous of difcovering fome medicine which might unite cheapnefs with efficacy. Recollecting the theoretical propofition of fome celebrated phyficians, that the epidemic dyfentery is an hæmorrhage of the inteftines brought on by animalculæ, I was led to imagine that the *nux vomica*, which is known to be fatal to large animals, might alfo prove equally deftructive to thefe animalculæ. The auftere tafte of this fubftance confirmed me in my ideas on this fubject, and knowing that it might be taken in fmall dofes without danger, I ventured to try its effects. I began by cleanfing the bowels with rhubarb and cream of tartar, after which I prefcribed a fcruple of nux vomica in in powder to be taken once a day. The good effects of this remedy exceeded my most sanguine expectations. To relate all the cases in which I administered it would be superfluous, I shall therefore content myself with giving the heads of the two following.

*Case* I. A boy fifteen years old was attacked with symptoms of fever, and violent griping pains in his bowels, attended with frequent bloody stools. He took every morning for some days a dose of rhubarb, and at night a dose of *Theriaca Andromachi*, but the dysenteric symptoms still remaining obstinate, I gave him a scruple of nux vomica once a day in barley water, and after taking four doses of it he was perfectly well.

*Case.* II. A tradesman in Linkoping after labouring for several days under a severe dysentery sent for me. As he was of a plethoric habit, and had a quick, full pulse, I began with prescribing venæsection, and a vomit of Ipecacuanha, after which I gave him rhubarb and cream of tartar, and at night a dose of Theriaca Andromachi. By this method he got better, but relapsing again, I administered the nux vomica, two doses of which proved effectual.

The good effects of this remedy in the above and a great number of other similar cases which fell fell under my own care, induced me to give a quantity of the powder to the Reverend Mr. Beckmark, Minister of Krisberg, to distribute it among his poor parishioners, but being afraid that they would not take it if they knew that it was *nux vomica*, which is considered as a poison, I gave it the name of *American Powder*. It was not long before the same clergyman applied to me for a fresh supply of the medicine, assuring me that it had had a wonderful good effect as well in the dysenteries that had succeeded a putrid fever, as in those in which no such fever had preceded. I received similar accounts from several other clergymen to whom I intrusted the same medicine for the use of the poor in their respective parishes. I have taken care to send copies of the letters I received from these Gentlemen to the College of Physicians at Stockholm, and likewise to the Chevalier Bacek, physician to the King. From these letters it appears that in each of these parishes a great number of dysenteric patients have been cured by this remedy; and that in the single parish of Schedwi it has been administered to 245 out of 255 persons who were attacked with dysentery, of which number only 22 died, ten of whom were children who refused to take the medicine, and the twelve twelve others were perſons who did not apply for relief till they were greatly reduced by the diſeaſe. In the greater number of caſes a cure was effected in three or four days. Many of the patients took the *nux vomica* alone without the previous uſe of rhubarb or any other medicine. Perſons who had been inured to hard labour and a coarſe diet were the ſubjects with whom it ſeemed to be moſt efficacious, and it was found to agree better and to be more active when taken in warm water or beer than when ſwallowed in either of thoſe liquors cold.

It is a known fact that oily and ſulphureous ſubſtances are deſtructive to *acari*, and I have met with ſeveral inſtances during the epidemic I have been ſpeaking of, where the dyſentery has been cured by ſweet butter, hog's lard, a mixture of gunpowder and brandy, &c.; and this obſervation ſeems to confirm the idea that theſe *animalculæ* are the cauſe of the violent griping in the dyſentery.

I ſhall reſerve my remarks on the effects of other medicines in this diſeaſe till another opportunity. In the mean time I ſhall hope that the remedy I have been recommending, and the utility of which I have ſo fully experienced, will be found equally efficacious by others.

